# Endometriosis in transmasculine individuals

**DOI:** 10.1530/RAF-21-0096

**Published:** 2022-04-20

**Authors:** Cecile A Ferrando

**Affiliations:** 1 Center for Urogynecology & Pelvic Reconstructive Surgery, Women’s Health Institute, Center for LGBT Care, Cleveland Clinic, Cleveland, Ohio, USA

## Abstract

Transmasculine people are assigned female at birth but identify as male. These patients often are prescribed testosterone therapy as part of their transition. This treatment can affect ovulation and stop menstrual periods. Endometriosis is a common condition that causes pelvic pain in some people born with female pelvic organs. Not a lot is known about transmasculine people and how often endometriosis affects them. Testosterone should help treat if not reduce the incidence of endometriosis. This commentary looks at the current literature in order to help clarify existing knowledge gaps. Transmasculine patients who present for hysterectomy as a surgery to help them affirm themselves in their self-identified gender sometimes report pelvic pain symptoms as well. There are many reasons why patients report pain before surgery, and this can be related to endometriosis, even though this diagnosis is less expected in this group. Providers caring for transmasculine patients should be aware of this.

Transmasculine individuals are assigned female at birth but some identify as male or choose to take on a masculine gender expression. Some patients choose to transition to their self-identified gender, and a proportion of these patients are prescribed long-term regimens of testosterone therapy. Testosterone therapy is known to cause cessation of ovulation and menses, and many of these patients report amenorrhea within months of starting testosterone. Very little data exist looking at the effects of short- and long-term testosterone use on ovarian and uterine function (MEDLINE Search 2000-2021). As clinicians treating this patient population, we have previously made assumptions about the low incidence of endometriosis in these patients; however, up until recently, very few publications have existed specifically looking at pelvic pain and endometriosis in transmasculine individuals and we do not know the prevalence of this condition in this patient population. This lack of knowledge can be attributed to the barriers many trans patients face when accessing care and their resultant underrepresentation in medicine. Therefore, the aim of this commentary is to review the literature on pelvic pain and endometriosis in transmasculine patients seeking hysterectomy.

Hysterectomy is a common gender-affirming surgery that some transmasculine patients undergo. Some transmasculine patients also present for management of pelvic pain and describe endometriosis symptoms. Sometimes, patients seek hysterectomy for a combination of their pain and their desire to transition to their self-identified gender. For some surgeons, it is hard to decipher the true indication for hysterectomy, but among those who routinely care for this patient population, it is widely accepted that hysterectomy is a reasonable choice for most patients, as the removal of the pelvic organs helps patients feel more aligned with their self-identified masculine gender, which reduces their gender dysphoria. In order to undergo hysterectomy as gender affirmation surgery, providers are encouraged to follow the World Professional Association for Transgender Health (WPATH) Standards of Care (WPATH 2021), which outline suggested preoperative requirements for patients and the providers caring for them.

As we know, endometriosis is most commonly diagnosed by visual inspection and identification of the disease. Specific to the transmasculine population, few studies have looked at and commented on the intraoperative findings of patients undergoing hysterectomy for gender affirmation, even in the setting of reported pelvic pain. In a study looking at adolescent patients, 35 transmasculine patients with mean age of 15 years presented with dysmenorrhea, and of those who were placed on testosterone therapy for gender affirmation, one in three had persistent pelvic pain symptoms (Shim *et al.* 2020). Of the patients in the cohort, seven were evaluated laparoscopically and all patients were found to have endometriosis. Our group at Cleveland Clinic recently published a retrospective analysis of 67 transmasculine individuals who underwent hysterectomy for gender affirmation (Ferrando *et al.* 2021). In this cohort, we found that only 60% of the patients were amenorrheic on testosterone and 50% reported pelvic pain at the time of the consultation. There was heterogeneity in the way that patients presented with gynecologic complaints in this cohort. All patients, however, desired hysterectomy to also treat their gender dysphoria. At the time of laparoscopy, the intraoperative incidence of endometriosis was 27%, and it was pathology-confirmed in 77.8% of cases. Endometriosis was found in 32% of patients who reported pelvic pain at the preoperative consultation and in 22% of patients who did not complain of pain. In addition, type of preoperative pelvic pain (constant vs cyclic) was not associated with endometriosis findings.

Very few pelvic organ pathology studies exist, looking at hysterectomy specimens in transgender men undergoing hysterectomy, and the reports that have been published show mixed findings. For example, Grimstad *et al.* (2019) described the characteristics of uterine pathology in 94 transgender men on testosterone who underwent hysterectomy for gender affirmation. Interestingly, the majority of the pathology reports from these patients demonstrated active endometrium. Conversely, Khalifa *et al.* (2019) looked at a similar patient population and reported that the majority of the specimens they evaluated had endometrial changes consistent with inactive endometrium. With these mixed findings, it is hard to definitively conclude what effects testosterone may have on the endometrium. But given that there is a report of the presence of active endometrium in some patients, we can consider that some patients do not have complete cessation of ovarian function and/or endometrial activity on testosterone, which means that in those patients predisposed to have endometriosis, even on testosterone therapy, they may have active disease.

The above finding could be attributed to aromatization of exogenous testosterone to estradiol in the peripheral tissues. There are no studies specifically looking at estradiol level trends in trans individuals using long-term testosterone. But it is very plausible that high levels of androgens may be converted to estrogen in this clinical scenario, leading to a hyper-estrogenic state in these patients. Endometriosis is considered an estrogen-dependent disease. And while the aforementioned hypothesis does not explain the mechanism by which endometriosis may occur in a higher proportion of trans individuals compared to their cisgender counterparts, it may explain why they may be symptomatic of their disease, even if they are amenorrheic.

As many as 1 in 10 women suffer from endometriosis. However, the prevalence of incidentally diagnosed disease is even higher. For instance, 17% of women undergoing laparoscopic ovarian drilling were found to have endometriosis at the time of their laparoscopic procedure (Hager *et al.* 2019), and in a large cohort of 465 women undergoing tubal sterilization, the reported incidence of an incidentally found endometriosis during these cases was nearly 12% (Tissot *et al.* 2017). In the study our group performed, we had paradoxical findings compared to what we see in our cisgender female population. We found that the incidence of incidental endometriosis was much higher than expected, but the incidence of endometriosis in patients who presented with complaints of pelvic pain preoperatively was much lower than what is commonly found in the cisgender women.

Endometriosis is present in up to 87% of patients who present for management of chronic pelvic pain (Falcone & Flyckt 2018). In our study, only one in three patients who presented with preoperative pelvic pain were found to have endometriosis at the time of their hysterectomy. In that paper, we describe the differences that exist between the transmasculine and cisgender female populations as it relates to perception of pain, which is a personal experience. In our paper, we write: ‘Transgender men carry the burden of feeling dysphoric about the mismatch between their biologic sex and their gender identity, which may manifest as pain or an amplification of the pain that is experienced with normal ovulation or menses. These patients may also be susceptible to high tone pelvic floor disorders although data on the incidence of this condition in transgender patients are sparse. Nevertheless, this type of pain should also be considered when counseling patients. Providers performing gender affirmation surgeries should be aware of these points and our recommendation is not to minimize or overlook the kind of pain that is felt by this patient population. While it may not always be endometriosis, persistent pain in these patients remains a potential indication for hysterectomy, and providers should give this consideration when evaluating this patient population’ (Ferrando *et al.* 2021). This finding highlights the complexity that exists in caring for this patient population. These patients may have less intraoperative findings in the setting of self-reported pain. Conversely, these patients tend to have a higher incidence of incidental findings of endometriosis, and so surgeons seeing these patients should consider this and be prepared to manage a complex pelvic case if necessary.

The majority of the studies that currently exist are limited by their retrospective nature and the reliance on the electronic medical record to obtain data. Because of these methods, we are not able to draw conclusions about pain characterization in this patient population. For instance, it is not clear if all patients presenting with pain have ‘endometriosis’-specific type pain, or whether it is a combination of this type of pain with other pain syndromes. Again, the contribution of the gender dysphoria felt by patients presenting for surgical interventions also makes it hard to interpret with confidence some of the results presented in these studies. Some patients may confuse what some cis women may describe as ‘normal’ menstrual discomfort with actual pelvic pain, as the discomfort felt about their bodies may amplify their symptoms. Future research should focus on better defining pain symptoms, collecting pre- and postoperative pain scores, and also creating comparative cohorts with cisgender populations seeking the same interventions for pain. This would allow us to better characterize pain symptoms in each group, to evaluate if pain is resolved in the same way and also to determine if endometriosis is similar between patient groups.

In summary, endometriosis exists in transmasculine individuals. Those seeking hysterectomy may present with reports of pain symptoms and some may only seek the procedure for gender affirmation. Either way, hysterectomy is a medically indicated procedure in individuals assigned female at birth seeking alignment with their self-identified masculine gender. In those with pain symptoms, providers should be aware that traditional management options may not always be appropriate for patients given their desire to virilize. Further, many patients feel favorable about having a hysterectomy for gender affirmation. Perhaps the newest piece of data we have now is that more patients than expected have incidental findings of endometriosis. Surgeons performing these procedures for this patient population should be aware of this and should be prepared in some cases to perform a more complicated hysterectomy.

## Declaration of interest

The author declares receiving authorship royalties from UpToDate, Inc.

## Funding

This work did not receive any specific grant from any funding agency in the public, commercial or not-for-profit sector.
